# Hemophagocytic Lymphohistiocytosis As the Initial Presentation of Occult Diffuse Large B-cell Lymphoma: A Diagnostic Challenge

**DOI:** 10.7759/cureus.115630

**Published:** 2026-09-02

**Authors:** Kavish Khatib, Shamnas Abdul-Aziz, Kashif Khatib

**Affiliations:** 1 Acute Medicine, Great Western Hospitals NHS Foundation Trust, Swindon, GBR; 2 Oncology, Chelsea and Westminster Hospital NHS Foundation Trust, London, GBR

**Keywords:** diffuse large b-cell lymphoma, hemophagocytic lymphohistiocytosis (hlh), hscore, hyperferritinemia, secondary hlh

## Abstract

Hemophagocytic lymphohistiocytosis (HLH) is a rare but life-threatening hyperinflammatory syndrome characterised by uncontrolled immune activation and cytokine release, resulting in multiorgan dysfunction and high mortality if not recognised and treated promptly. In adults, secondary HLH is frequently triggered by infection, autoimmune disease, or malignancy, with haematological malignancies carrying a particularly poor prognosis. The non-specific nature of its presentation often leads to diagnostic delay, as it can closely mimic sepsis and other inflammatory conditions.

We present the case of a 76-year-old man who was admitted with a two-week history of progressive lethargy, exertional dyspnoea, anorexia, and headache after several months of constitutional symptoms. On presentation, he was hypotensive and tachycardic with atrial fibrillation and was initially managed for presumed sepsis with associated haemodynamic compromise. Despite supportive management, his clinical condition and biochemical profile deteriorated, with worsening cytopenias, deranged liver function tests, persistent hyperlactataemia, pyrexia, and escalating inflammatory markers. Further investigation demonstrated marked hyperferritinaemia exceeding 10,000 µg/L, hypertriglyceridaemia, hypofibrinogenaemia, elevated lactate dehydrogenase, and bicytopenia, producing an HScore of 220, which was highly suggestive of secondary HLH.

Following multidisciplinary discussion with regional specialists, treatment with high-dose IV methylprednisolone and anakinra was commenced while an urgent search for the underlying trigger continued. Initial imaging failed to identify a clear source of malignancy or infection, and the patient showed only limited improvement with HLH-directed immunomodulatory therapy. Bone marrow biopsy subsequently demonstrated high-grade diffuse large B-cell lymphoma (DLBCL) with marrow involvement, establishing malignancy-associated HLH as the underlying diagnosis. The patient was transferred to a specialist haematology service and commenced on R-mini-CHOP chemotherapy, resulting in rapid clinical recovery and marked improvement in HLH parameters. Subsequent positron emission tomography imaging demonstrated a complete metabolic response after two treatment cycles, with the patient improving from a bedbound state to being independently ambulatory.

This case highlights the significant diagnostic challenges associated with adult HLH and emphasises the importance of maintaining a high index of suspicion in patients presenting with unexplained fever, cytopenias, hyperferritinaemia, liver dysfunction, and multiorgan inflammation. It also demonstrates that, while immunosuppressive therapy may provide temporary disease control, successful management of malignancy-associated HLH depends on prompt identification and definitive treatment of the underlying haematological malignancy. Early multidisciplinary collaboration and timely bone marrow examination are critical to improving outcomes in this rapidly progressive and frequently fatal condition.

## Introduction

Hemophagocytic lymphohistiocytosis (HLH) is a rare but life-threatening hyperinflammatory syndrome characterised by uncontrolled activation of macrophages, cytotoxic T lymphocytes, and natural killer cells, resulting in excessive cytokine release, multiorgan dysfunction, and high mortality if not recognised promptly [1]. Although primary HLH is predominantly a paediatric disorder associated with inherited defects in immune regulation, adult HLH is most commonly secondary to infection, autoimmune disease, or malignancy [2,3]. Among adults, haematological malignancies, particularly aggressive lymphomas, represent some of the most frequent triggers and are associated with particularly poor clinical outcomes [3].

The diagnosis of HLH in adults remains challenging because its clinical manifestations are non-specific and frequently overlap with severe sepsis and systemic inflammatory response syndrome [2,4]. Common features include persistent fever, cytopenias, hyperferritinaemia, hypertriglyceridaemia, hypofibrinogenaemia, liver dysfunction, and coagulopathy, although these abnormalities often develop progressively rather than simultaneously [1,5]. Diagnostic tools such as the HLH-2004 criteria and the HScore have improved recognition of the syndrome; however, early clinical suspicion remains essential, as delays in diagnosis and treatment are associated with rapid deterioration and significant mortality [1,5,6]. The HLH-2004 criteria were primarily developed in paediatric populations and may have limited applicability in adult secondary HLH, while the HScore, although useful in adults, is not disease-specific and should be interpreted alongside clinical judgement and the overall clinical context rather than as a standalone diagnostic criterion.

Malignancy-associated HLH is a distinct clinical entity in which the cytokine storm is driven by the underlying neoplasm. Diffuse large B-cell lymphoma (DLBCL), while the most common subtype of non-Hodgkin lymphoma, is an uncommon but well-recognised cause of secondary HLH and may present without obvious lymphadenopathy or radiological evidence of malignancy, making diagnosis particularly challenging [2,3]. In these patients, immunomodulatory therapy alone is often insufficient, and successful control of the hyperinflammatory state depends upon prompt identification and definitive treatment of the underlying lymphoma [6].

We present the case of a 76-year-old man who presented with constitutional symptoms, atrial fibrillation with rapid ventricular response, hypotension, and biochemical features initially suggestive of severe sepsis. Progressive cytopenias, marked hyperferritinaemia, hypertriglyceridaemia, hypofibrinogenaemia, and worsening multiorgan dysfunction led to the diagnosis of secondary HLH, with subsequent bone marrow biopsy revealing previously occult high-grade DLBCL. This case highlights the diagnostic complexity of adult HLH and reinforces the importance of early multidisciplinary investigation and aggressive evaluation for an underlying haematological malignancy.

## Case presentation

A 76-year-old man with a medical history of atrial fibrillation status post catheter ablation, ischaemic heart disease, mild tricuspid regurgitation, Raynaud’s phenomenon undergoing investigation for a possible connective tissue disorder, and glaucoma presented with a two-week history of progressive lethargy, exertional dyspnoea, anorexia, and persistent headache. He also described several months of constitutional symptoms following a flu-like illness, including worsening Raynaud’s symptoms and chilblain-like lesions affecting his feet. His general practitioner noted hypotension and tachycardia and referred him for urgent same-day assessment.

On admission, the patient appeared pale and lethargic but remained alert and orientated. His observations demonstrated atrial fibrillation with a rapid ventricular response (heart rate 133 beats/min), blood pressure 99/61 mmHg, respiratory rate 24 breaths/min, oxygen saturation 99% on room air, and temperature 36.8°C. Physical examination revealed delayed peripheral capillary refill and dry mucous membranes but was otherwise unremarkable, with no focal neurological deficit, meningism, abdominal tenderness, peripheral lymphadenopathy, or clinically appreciable hepatosplenomegaly.

**Figure 1:**
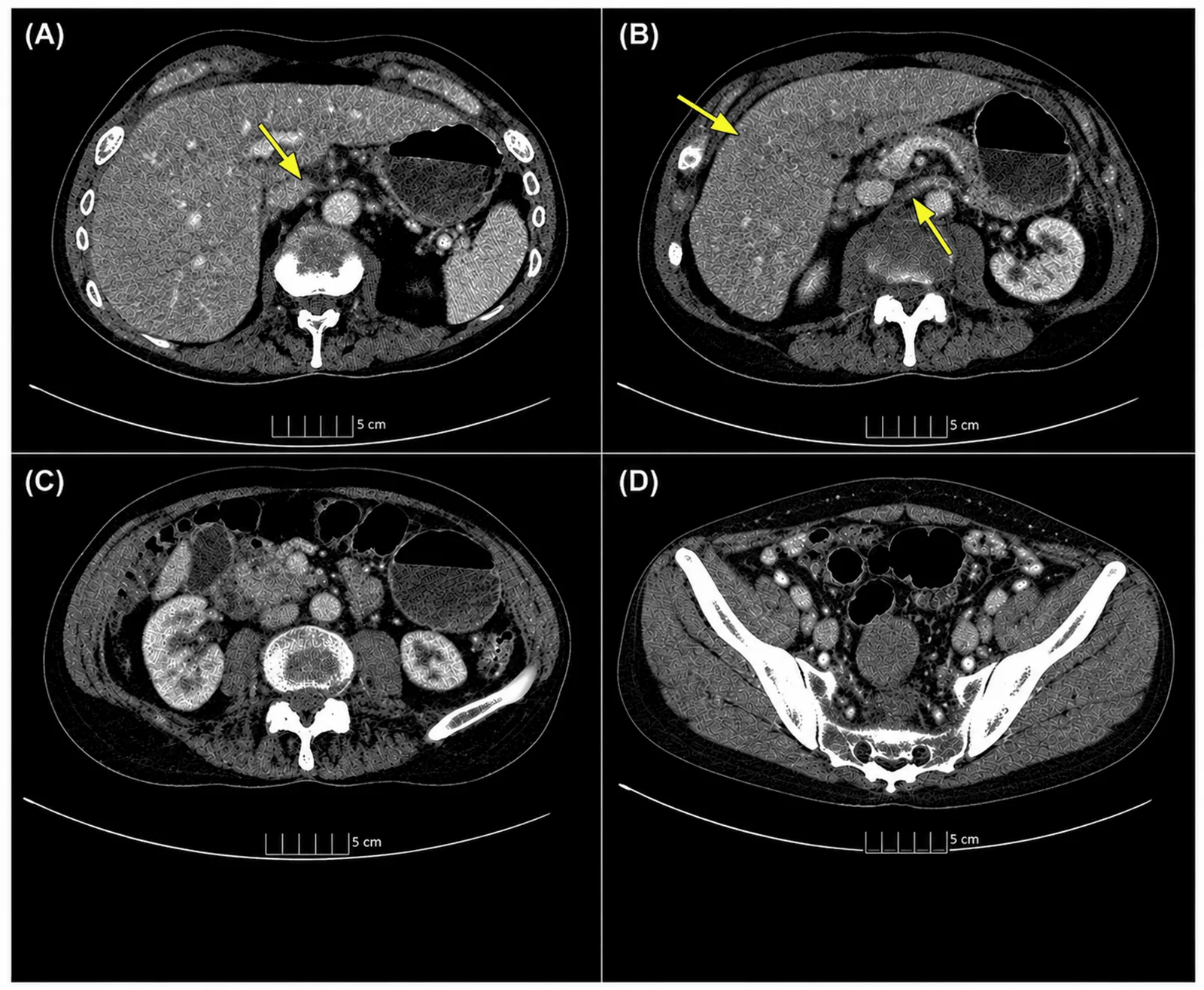
Contrast-enhanced CT of the abdomen and pelvis at initial presentation. (A, B) Axial contrast-enhanced CT images of the upper abdomen demonstrate mild inflammatory fat stranding surrounding the pancreatic head (arrow), with subtle periportal oedema within the left hepatic lobe (arrow). (C) Axial image through the lower abdomen demonstrates no additional acute intra-abdominal abnormality. (D) Axial pelvic image demonstrates no pelvic lymphadenopathy. There is no hepatomegaly, splenomegaly, biliary dilatation, radiopaque gallstones, or radiological evidence of acute cholecystitis.

Initial laboratory investigations (Table 1) demonstrated elevated inflammatory markers (C-reactive protein 111 mg/L), cholestatic liver function abnormalities (bilirubin 37 µmol/L, alkaline phosphatase 293 U/L, alanine aminotransferase 55 U/L), and a markedly elevated venous lactate of 7.3 mmol/L despite preserved renal function. Electrocardiography confirmed atrial fibrillation with a rapid ventricular response. Chest radiography showed no acute cardiopulmonary abnormality. Computed tomography of the abdomen and pelvis (Figure 1) demonstrated mild periportal oedema within the liver and non-specific inflammatory changes around the pancreatic head but no convincing intra-abdominal source of sepsis, lymphadenopathy, or hepatosplenomegaly.

**Table 1:** Laboratory investigations on admission and at the time of diagnosis of hemophagocytic lymphohistiocytosis (HLH). Comparison of key laboratory parameters at hospital admission and at the time the patient fulfilled the diagnostic criteria for HLH, demonstrating progression to hyperinflammation, including marked hyperferritinaemia, hypertriglyceridaemia, hypofibrinogenaemia, worsening thrombocytopenia, and liver dysfunction. ↑ indicates a value above the reference range; ↓ indicates a value below the reference range. The earliest available fibrinogen measurement was within the reference range before subsequently declining to 1.2 g/L during the evolution of HLH. HLH: Hemophagocytic lymphohistiocytosis; ALT: Alanine aminotransferase; AST: Aspartate aminotransferase; ALP: Alkaline phosphatase; IU/L: International units per litre; g/L: Grams per litre; µg/L: Micrograms per litre; mmol/L: Millimoles per litre; ×10⁹/L: ×10⁹ cells per litre.

Investigation	Reference range	At admission	At HLH diagnosis (day 2 of admission)
Haemoglobin (g/L)	130-170	131	112 ↓
White cell count (×10⁹/L)	3.7-11.0	4.82	5.05
Platelets (×10⁹/L)	150-400	71 ↓	26 ↓
Ferritin (µg/L)	30-336	2,199 ↑	10,866 ↑
Triglycerides (mmol/L)	<1.7	-	7.46 ↑
Fibrinogen (g/L)	2.3-4.7	4.3*	1.2 ↓
C-reactive protein (mg/L)	0-5	111 ↑	49 ↑
ALT (IU/L)	<50	55 ↑	272 ↑
AST (IU/L)	<50	-	320 ↑
ALP (IU/L)	30-130	293 ↑	1,091 ↑
Bilirubin (µmol/L)	0-21	37 ↑	55 ↑
*Earliest available fibrinogen measurement was within the reference range before subsequently falling to a nadir of 1.2 g/L during the development of HLH.			

The patient was initially managed for presumed sepsis with secondary atrial fibrillation and haemodynamic compromise. Intravenous fluid resuscitation and digoxin were administered for rate control, and he was admitted for further investigation. Despite supportive management, his clinical condition progressively deteriorated over the following days, with persistent pyrexia, worsening thrombocytopenia and leucopenia, progressive liver dysfunction, and persistently elevated inflammatory markers. The combination of unexplained cytopenias and ongoing clinical deterioration prompted further investigation for a hyperinflammatory syndrome.

Additional laboratory testing (Table 1) demonstrated a serum ferritin concentration greater than 10,000 µg/L, triglycerides of 7.46 mmol/L, hypofibrinogenaemia (1.2 g/L), markedly elevated lactate dehydrogenase, and bicytopenia. The patient fulfilled five of the eight HLH-2004 diagnostic criteria; soluble CD25 levels and NK-cell activity were not assessed due to the limited availability of these specialised tests. Collectively, these findings generated an HScore of 220, indicating a very high probability of secondary HLH. Following multidisciplinary discussion with rheumatology and haematology specialists, empirical treatment with intravenous methylprednisolone was initiated, followed by anakinra, while investigations for the underlying trigger continued.

Extensive microbiological and autoimmune investigations failed to identify an alternative infective or rheumatological cause for the patient's hyperinflammatory state. Bone marrow aspiration and trephine biopsy were subsequently performed. Although the initial aspirate was insufficient for definitive analysis, subsequent histopathological examination demonstrated high-grade DLBCL involving the bone marrow, supporting lymphoma-associated secondary HLH as the predominant diagnosis. During the diagnostic workup, CMV DNA was also detected by qualitative PCR; however, a quantitative viral load was not available. Given the possibility of CMV viraemia representing a contributory infectious trigger for HLH, ganciclovir was commenced following specialist advice. The subsequent identification of high-grade DLBCL with bone marrow involvement, together with the limited response to initial HLH-directed and antiviral therapy and the marked clinical and biochemical improvement following lymphoma-directed chemotherapy, supported DLBCL as the predominant underlying driver of HLH, although a contributory role of CMV could not be excluded.

Following confirmation of the diagnosis, the patient was transferred to the regional haematology service and commenced on R-mini-CHOP chemotherapy. Clinical and biochemical improvement occurred rapidly after initiation of lymphoma-directed treatment, with progressive resolution of cytopenias, reduction in inflammatory markers, and normalisation of HLH parameters. Interim positron emission tomography-computed tomography performed after two cycles demonstrated a complete metabolic response. The patient's functional recovery was remarkable, improving from a bedbound performance status at diagnosis to independent ambulation and a return to regular physical activity. The treatment plan included completion of six cycles of R-mini-CHOP, selected in view of the patient’s frailty and poor performance status following severe HLH, to balance effective lymphoma-directed treatment against the risk of treatment-related toxicity. This was to be followed by repeat bone marrow biopsy and interval imaging to confirm sustained remission.

## Discussion

HLH is a rare but rapidly progressive hyperinflammatory syndrome with a reported mortality exceeding 40% in adults despite advances in treatment [2,3]. Prompt diagnosis remains one of the greatest clinical challenges because patients frequently present with non-specific symptoms, including fever, constitutional symptoms, cytopenias, liver dysfunction, and multiorgan failure, all of which closely resemble severe sepsis or systemic inflammatory response syndrome [2,4]. Consequently, delays in recognition are common and contribute significantly to adverse outcomes.

Our patient initially presented with constitutional symptoms, atrial fibrillation with rapid ventricular response, hypotension, elevated inflammatory markers, deranged liver function tests, and hyperlactataemia, all of which suggested an infective process. However, progressive bicytopenia, worsening biochemical abnormalities, and marked hyperferritinaemia prompted reconsideration of the diagnosis. Hyperferritinaemia is a hallmark feature of HLH, and although elevated ferritin lacks specificity, values exceeding 10,000 µg/L should strongly prompt consideration of HLH in the appropriate clinical context [1,5]. Similarly, the coexistence of hypertriglyceridaemia, hypofibrinogenaemia, and cytopenias reflected the profound immune dysregulation characteristic of this syndrome.

The HScore has become an important diagnostic tool for adult HLH and incorporates clinical, laboratory, and bone marrow findings to estimate the probability of reactive hemophagocytic syndrome [5]. In this case, the patient's HScore of 220 indicated a very high probability of HLH and supported the decision to initiate treatment before histological confirmation. This illustrates an important clinical principle: because untreated HLH can progress rapidly to irreversible multiorgan failure, therapy should not necessarily be delayed while awaiting definitive tissue diagnosis when clinical suspicion is high [3].

Adult HLH is most commonly secondary to infection, autoimmune disease, or malignancy, with haematological malignancies accounting for a substantial proportion of cases [2,3]. Lymphoma-associated HLH is recognised as one of the most aggressive forms of secondary HLH and is associated with particularly poor survival [6]. Although T-cell and natural killer-cell lymphomas are classically implicated, DLBCL is an increasingly recognised trigger and may present without obvious lymphadenopathy or radiological abnormalities, making diagnosis difficult [6]. In our patient, initial imaging failed to identify a clear malignant process, emphasising the limitations of conventional imaging and the importance of bone marrow examination in patients with suspected malignancy-associated HLH.

The patient received high-dose methylprednisolone followed by anakinra after multidisciplinary discussion. Corticosteroids remain a cornerstone of initial HLH management because they suppress excessive immune activation, while interleukin-1 blockade with anakinra has emerged as an effective therapeutic option, particularly in secondary HLH and macrophage activation syndrome [3,7]. Nevertheless, our patient demonstrated only a limited response to immunomodulatory therapy, highlighting that treatment directed solely at the inflammatory cascade may be insufficient when an underlying malignancy remains active.

Definitive clinical improvement occurred only after initiation of R-mini-CHOP chemotherapy following confirmation of high-grade DLBCL on bone marrow biopsy. This rapid resolution of inflammatory markers and recovery in functional status strongly support the concept that successful treatment of malignancy-associated HLH depends on eradication of the underlying trigger rather than prolonged immunosuppression alone [3,6]. The patient's rapid improvement from a bedbound performance status to complete metabolic remission after chemotherapy further illustrates the reversibility of even severe HLH when appropriate disease-specific treatment is instituted promptly, consistent with published systematic reviews [8].

This case highlights several important learning points for acute physicians and haematologists. First, HLH should be considered in any patient with unexplained fever or systemic inflammation accompanied by cytopenias, liver dysfunction, and markedly elevated ferritin concentrations, particularly when the clinical course is disproportionate to the presumed infection. Second, the HScore provides a valuable adjunct to clinical judgement and may facilitate earlier recognition and treatment. Finally, aggressive investigation for occult haematological malignancy, including early bone marrow biopsy when appropriate, is essential because definitive treatment of the underlying lymphoma may be a key determinant of survival in malignancy-associated HLH.

Further awareness of adult HLH among acute care physicians is needed to reduce diagnostic delay and improve patient outcomes. Early multidisciplinary collaboration involving acute medicine, haematology, rheumatology, and intensive care teams is often required, and prompt initiation of both HLH-directed therapy and treatment of the underlying trigger offers the greatest opportunity for survival in this rare but devastating syndrome. HLH and severe sepsis share many overlapping clinical and laboratory features, and failure to recognise this important differential diagnosis may delay definitive treatment and adversely affect prognosis [9]. Adult HLH remains associated with substantial mortality and requires prompt recognition and multidisciplinary management to improve outcomes [10,11].

## Conclusions

HLH is a rare but life-threatening hyperinflammatory syndrome that may present with non-specific features resembling severe sepsis, making diagnosis particularly challenging. This case demonstrates that secondary HLH can be the initial manifestation of otherwise occult DLBCL. In adults presenting with unexplained fever, cytopenias, hyperferritinaemia, hypertriglyceridaemia, hypofibrinogenaemia, and progressive organ dysfunction despite appropriate treatment, HLH should be considered early, and structured diagnostic tools such as the HScore may support timely recognition.

This patient's clinical recovery following treatment with corticosteroids, anakinra, and ultimately R-mini-CHOP chemotherapy highlights that successful management of malignancy-associated HLH depends on prompt identification and treatment of the underlying malignancy. Early multidisciplinary collaboration and a high index of suspicion are essential to minimise diagnostic delay, initiate appropriate therapy, and improve clinical outcomes in this potentially fatal condition.
